# Gambling in the Visual Periphery: A Conjoint-Measurement Analysis of Human Ability to Judge Visual Uncertainty

**DOI:** 10.1371/journal.pcbi.1001023

**Published:** 2010-12-02

**Authors:** Hang Zhang, Camille Morvan, Laurence T. Maloney

**Affiliations:** 1 Department of Psychology, New York University, New York, New York, United States of America; 2 Center for Neural Science, New York University, New York, New York, United States of America; 3 Department of Psychology, Harvard University, Cambridge, Massachusetts, United States of America; University College London, United Kingdom

## Abstract

Recent work in motor control demonstrates that humans take their own motor uncertainty into account, adjusting the timing and goals of movement so as to maximize expected gain. Visual sensitivity varies dramatically with retinal location and target, and models of optimal visual search typically assume that the visual system takes retinal inhomogeneity into account in planning eye movements. Such models can then use the entire retina rather than just the fovea to speed search. Using a simple decision task, we evaluated human ability to compensate for retinal inhomogeneity. We first measured observers' sensitivity for targets, varying contrast and eccentricity. Observers then repeatedly chose between targets differing in eccentricity and contrast, selecting the one they would prefer to attempt: e.g., a low contrast target at 2° versus a high contrast target at 10°. Observers knew they would later attempt some of their chosen targets and receive rewards for correct classifications. We evaluated performance in three ways. **Equivalence**: Do observers' judgments agree with their actual performance? Do they correctly trade off eccentricity and contrast and select the more discriminable target in each pair? **Transitivity**: Are observers' choices self-consistent? **Dominance**: Do observers understand that increased contrast improves performance? Decreased eccentricity? All observers exhibited patterned failures of equivalence, and seven out of eight observers failed transitivity. There were significant but small failures of dominance. All these failures together reduced their winnings by 10%–18%.

## Author Summary

Human ability to discriminate drops dramatically with increasing distance from the center of vision. If you fixate a word on a page, you likely can not read words a short distance away. Because of this retinal inhomogeneity, we need to move our eyes to search a scene. The efficiency of search depends on how well the visual system compensates for inhomogeneity in planning eye movements. We used a simple decision task to find out what the observer “knows” about his or her own retina. We first measured observers' sensitivity for targets, varying contrast and eccentricity. Observers then repeatedly chose between targets differing in eccentricity and contrast, selecting the one they would prefer to attempt: e.g., a low contrast target at 2° versus a high contrast target at 10°. Could observers correctly trade off contrast and eccentricity and pick the more discriminable of the two targets? We found that observers exhibited large, patterned errors in their choices, making choices that were not even self-consistent.

## Introduction

**Figure 1:**
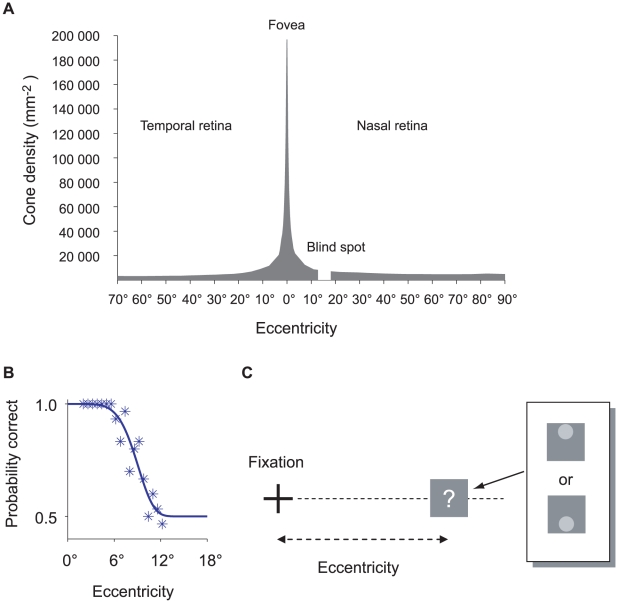
Retinal inhomogeneity. **A. The density of cone photoreceptors in the human retina.** The density of cones is the highest at the fovea and drops sharply with increasing eccentricity. Plotted data from Curcio et al. [1]. Eccentricity in millimeter was transformed into degree using Drasdo and Fowler's [44] curve for retinal eccentricity and areal magnification. **B. Retinal scaling curve for one contrast and one observer.** The retinal scaling curve is a plot of probability of correct response as a function of retinal eccentricity in degrees of visual angle. Near the fovea the observer is consistently correct while beyond 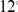 the observer is at chance. **C. The calibration task.** On each trial, one of two configurations (inset) was displayed. The observer's task was to judge which of the two configurations was presented. The contrast and retinal location (eccentricity) varied from trial to trial.

An average human eye has as many as 4.6 million cones in a retinal area of 1019 mm^2^ [1] centered on the fovea but the distribution of cones across the retina is far from uniform (Figure 1A). As a consequence of retinal inhomogeneity and post-receptoral processing [2], observer's performance in psychophysical tasks can vary markedly with retinal eccentricity. This variation can be summarized by a retinal sensitivity curve such as the one shown in Figure 1B.

The retinal sensitivity curve in Figure 1B is a plot of the probability of correct discrimination as a function of retinal eccentricity. The observer attempted to discriminate two possible configural targets (Figure 1C) consisting of a small circle superimposed on a square. As shown, the observer's probability of correct discrimination is close to one when the target is near the fovea and drops to chance beyond 12 degrees.

Retinal scaling curves for many kinds of visual judgments have been measured [3]–[6] and researchers modeling visual search typically assume that the visual system effectively has access to estimates of visual sensitivity for different kinds of targets at different eccentricities [7]–[10]. This information is needed to correctly combine visual data from disparate retinal locations, detect the target or plan the next saccade. Since the mapping between eccentricity and visual sensitivity may differ for different kinds of targets, the amount of information needed to plan visual search well is potentially very large.

We examined whether human observers have access to this information in a simple decision task. In the first part of the experiment (*calibration*) we mapped retinal scaling curves for the configural target at three contrasts, High, Medium and Low. Targets were placed along a horizontal line passing through the fovea and each target could be thought of as an ordered pair 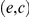 where 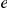 is horizontal distance from the fovea, 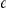 is contrast.

In the main part of the experiment (*decision*), the observers were asked to judge which of two configural targets, differing in contrast and in retinal eccentricity, 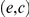 or 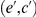, was more discriminable. A judgment that 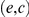 is/was more discriminable than 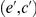 is denoted 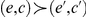. Observers knew that, at the end of the experiment, they would be allowed to attempt to classify some of the targets they had chosen, receiving a reward for each correct response. It was therefore in their interest to select the more discriminable eccentricity-contrast pair on each trial.

Unlike typical decision tasks [11], this decision task does not involve a tradeoff between probability and value: we never varied the payoffs for success and failure. Successful performance requires only that the observer correctly orders probabilities. Performance would also be unaffected by monotone increasing transformations of probability commonly reported in the decision under risk literature [11]–[13].

The decision task is an example of a conjoint measurement task [14]–[15]. We vary contrast and eccentricity and see how these variations affect the observer's ordering of eccentricity-contrast pairs 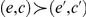 by discriminability. If the observer's judgments satisfy certain conditions that, in effect, assess their coherence or self-consistency, then the experimenter can potentially recover estimates of the observer's “subjective” retinal sensitivity curves for each contrast [14]–[15] and compare them to observers' actual performance. If one or more of the conditions fail, then we further conclude that the observer's choices are not based on a coherent model of their own retinal sensitivity.

**Figure 2:**
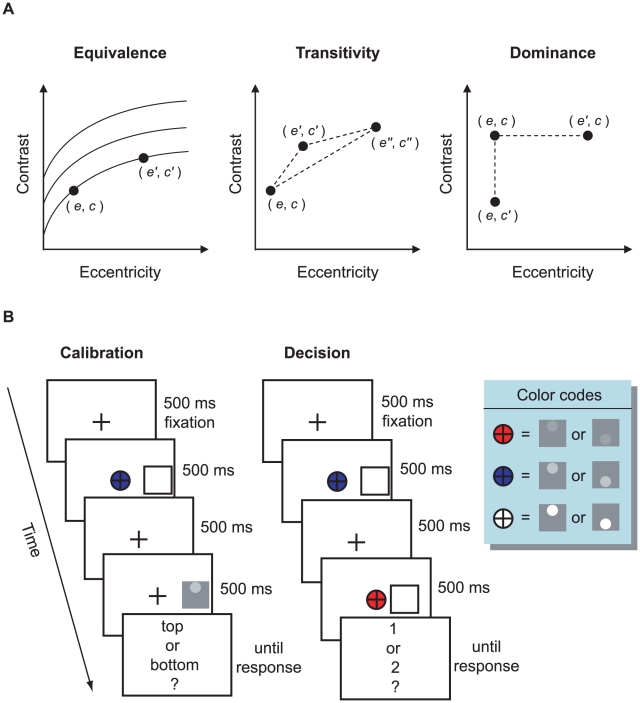
Methods. **A. Conjoint measurement: testing equivalence, transitivity, and dominance.** In testing equivalence we used data from the calibration phase of the experiment to compare the actual discriminability of eccentricity-contrast pairs that observers judged to be equally discriminable. Observers could err in selecting equally discriminable pairs but still make judgments that were self-consistent. The contours shown are examples of contours of equal discriminability. We tested self-consistency by testing transitivity. See text. The test of dominance evaluates whether observers understood that, all else equal, higher contrast or lower eccentricity led to better performance. See text. **B. Time courses of the calibration and decision tasks.** In the calibration task we measured retinal scaling curves for three contrasts. The observer learned to associate each of the three contrasts with a color code (inset). In the decision task, the contrast of each 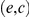 pair was signaled using color codes. Note that the target contrasts in the inset legend are just for illustration and are considerably higher than those used in the experiment.

We test the observer's knowledge of his own ability to discriminate such targets in three ways, illustrated in Figure 2A. The first is a test of *equivalence*: Can observers correctly judge which pairs 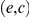 and 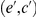 are equally discriminable? We can represent these pairs by indifference curves as shown in Figure 2A.

The second test is *transitivity*: for any choice of eccentricities 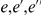 and contrasts 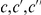, if 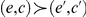 and 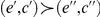, then 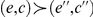. Transitivity is a test of the self-consistency or coherence of observers' judgments.

The third is a test of *dominance*: if 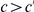, does the observer correctly judge that 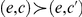 for any choice of eccentricity 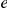
? And if 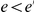, does the observer correctly judge that 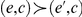 for any choice of contrast 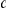
? (Of course, we must verify experimentally that the two dominance claims are in fact true for our experimental conditions). Dominance is evidently the weakest of the three tests.

The three tests are distinct: an observer who fails equivalence may still satisfy transitivity and dominance. This outcome would imply that, while his or her estimates of discriminability are in error, the estimates he or she has do, at least, cohere. An observer who fails transitivity cannot trade off contrast and eccentricity in any consistent way, but he or she may still know that more contrast improves performance and that performance near the fovea is better.

## Methods

### Ethic statement

The experiment had been approved by the University Committee on Activities Involving Human Subjects (UCAIHS) of New York University and informed consent was given by the observer prior to the experiment.

### Apparatus

Stimuli were displayed on a 19-in. Sony Trinitron Multiscan G500 monitor controlled by a Dell Pentium D Optiplex 745 computer. The monitor was run at a frame rate of 100 Hz with 1280×1024 resolution in pixels. A forehead bar and chinrest were used to help the observer maintain a viewing distance of 57 cm. At that distance, the full display subtended 40.4°×30.3°. The observer viewed the display binocularly.

### Monitoring fixation

Observers were required to fixate a fixation cross and all stimuli were presented relative to this fixation cross. We used an Eyelink II eye tracker to verify that observers did not make eye movements away from the fixation cross. At the beginning of each trial drift correction was made at the fixation cross. The criterion of eye movement was set to be a speed over 10 deg/s or an offset over 1 deg from the fixation cross. A trial would be cancelled if the fixation constraint were violated during the trial. The eye tracker was calibrated initially, drift corrected for each trail and re-calibrated after every 100 trials or when drift exceeded 5 deg.

### Stimuli

Stimuli were presented against a uniform gray (39.1 cd/m^2^) background. The fixation cross was black, spanning 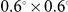 at the center of the screen. The target was a 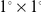 lighter gray (67.1 cd/m^2^) square with an even lighter gray dot of 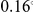 diameter at its top or bottom. The luminance of the dot could be 74.4, 80.7, or 91.4 cd/m^2^, i.e., a contrast of 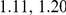 or 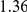 relative to the square. We refer these three levels of contrast as low, medium, and high contrast. The contrast 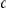 of a stimulus and the eccentricity at which it was presented, formed an *eccentricity-contrast pair* 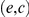.

#### Color codes and location cues

Each contrast was associated with a colored cue, which was a filled circle of 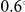 diameter behind the fixation cross. The colors for the low, medium, and high contrast were red, blue and white, respectively. The location of target was cued by a 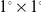
black frame square at the location of the would-be target. Targets or location cues were located at 18 possible locations uniformly distributed in the range of 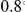 to 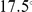 to the right of the fixation cross.

### Procedure and design

The experiment consisted of two three-hour sessions completed on two successive days. Observers were advised to take a break every about 350 trials and allowed to take breaks whenever necessary. Each observer went through the two tasks in sequence: calibration, then decision. The time courses of both tasks are illustrated in Figure 2B.

### Calibration task

The calibration task allowed us to map probability correct as a function of eccentricity for each of the three contrasts. The observer's task was to decide whether the dot was at the top or at the bottom (Figure 2B). Fixation was monitored. No feedback was given.

For each of the three contrasts, the target could appear at any of 18 possible locations, i.e., 18 possible eccentricities, evenly spaced from 2° to 12.2° on the right of the fixation cross. There were five blocks, in each of which each location of each contrast repeated for six times, half top and half bottom, randomly mixed together. Each observer completed 3 contrasts×540 trials = 1620 calibration trials.

Before the experimental trials, there were 108 practice trials for the first session and 12 practice trials for the second session. To keep observers motivated, we rewarded observers with a bonus up to $10 based on their overall probability correct in the calibration task of each session.

The probability correct of the calibration task was fitted against eccentricity with a Quick-Weibull psychometric function [16]–[17]:
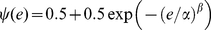
(1)where 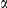 is a position parameter and 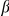 is a steepness parameter. With Equation 1, we could compute the probability correct for any eccentricity. We used these functions in the construction of decision trials.

### Decision task

We described the decision task in the Introduction. In this task, observers chose between two targets of different combinations of contrast and retinal eccentricity. But rather than using real targets, we used a location cue and a color cue to indicate an eccentricity-contrast pair. The observer's task was to choose the target they preferred to attempt later (Figure 2B). As in the calibration task, observers were required to fixate the fixation cross before the response display.

There were two reasons why we did not use real targets to signify eccentricity-contrast pairs. First, if observers had seen real targets in a trial, they could base their decision on their immediate perception of the targets, in effect simulating the visual judgment. Second, with real targets, observers might mistake one contrast condition for another.

Observers learned the association between targets and cues at the beginning of the experiment during the calibration task and we verified that they had learned these associations by a short “quiz” before the decision task.

Observers knew that, at the end of the experiment, four of their choices would be chosen at random and that they would attempt to identify targets in the conditions corresponding to each choice. A correct response would lead to a $5 reward. Correct response for all of the four trials would result in a $20 bonus.

To measure the point of subjective indifference (equivalence) between targets that differed in contrast, we used one-up, one-down adaptive staircase procedures. In a staircase, one target of one contrast was fixed in eccentricity and the target of the other contrast varied in eccentricity. The fixed contrast in each staircase had an eccentricity corresponding to a probability correct of 0.6, 0.7, 0.8, or 0.9 separately estimated for each observer based on their calibration data. We estimated the eccentricity that the observer considered to be equally discriminable for the variable contrast. Each staircase consisted of 70 trials. There were 12 staircases (3 contrasts×4 probabilities), randomly interleaved. That is, 12 staircases×70 trials = 840 staircase trials. Based on these staircase trials, we tested equivalence and transitivity.

To test dominance, we included trials in which the two targets had different eccentricities but the same contrast (*equi-contrast trials*), or different contrasts but the same eccentricity (*equi-eccentricity trials*). The possible contrasts were low, medium, and high. The possible eccentricities were the eccentricities corresponding to a probability correct of 0.75 for each of the three contrasts, computed with the functions estimated in the calibration task for the particular observer. The number of equi-contrast trials was 3 contrasts×3 eccentricity combinations×10 repetitions = 90. The number of equi-eccentricity trials was 3 eccentricities×3 contrast combinations×10 repetitions = 90 as well.

The 840 staircase trials and 180 dominance trials were mixed in a random order, divided into three blocks and completed in the second session after the calibration task. There were 24 practice trials before the formal experimental trials.

### Observers

Eight observers, four female and four male, participated. None of them was aware of the purpose of the experiment. All observers had normal or corrected-to-normal vision. The observers each received US $12/hour for their time and a performance-related bonus. Total payment ranged from US $87 to US $112 across observers.

## Results

### Visual sensitivity curves

**Figure 3:**
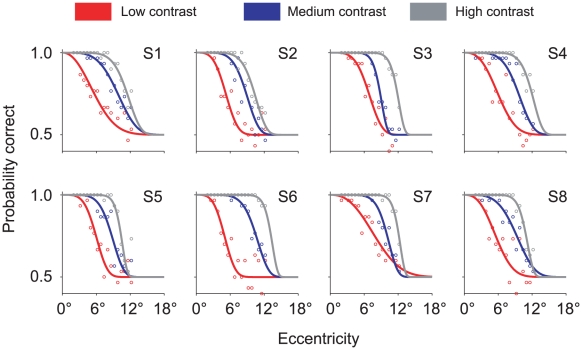
Calibration task: Visual sensitivity curves. Probability of correct response is plotted against retinal eccentricity for each of three contrasts. Each panel corresponds to one observer. Circles denote data. Solid lines denote the fits of Equation 1 to data. Red, blue, and gray respectively denote low, medium, and high contrast.

For each observer, we fit the data of the calibration task to Equation 1 separately for each contrast using the maximum likelihood method. Figure 3 shows both the data and fit for each observer.

### Equivalence test

From the 12 staircases of the decision task, we acquired 12 pairs of eccentricity-contrast pairs judged to be equally discriminable 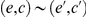 by the observer. Four of them had the target of low contrast in fixed eccentricity and the target of medium contrast in variable eccentricity, which we call a low-to-medium mapping. Another four staircases were medium-to-high mappings and a third set of four high-to-low mappings.

For each observer, we computed the differences between the actual probabilities correct of the fixed and variable targets (based on calibration data) and examined whether they significantly deviated from zero. We used a bootstrap method [18] to estimate 95% confidence intervals for the probability difference of each pair for each observer (10,000 resamples). We tested whether differences in probability were significant at an overall level of .05 with a Bonferroni correction for 12 tests.

**Figure 4:**
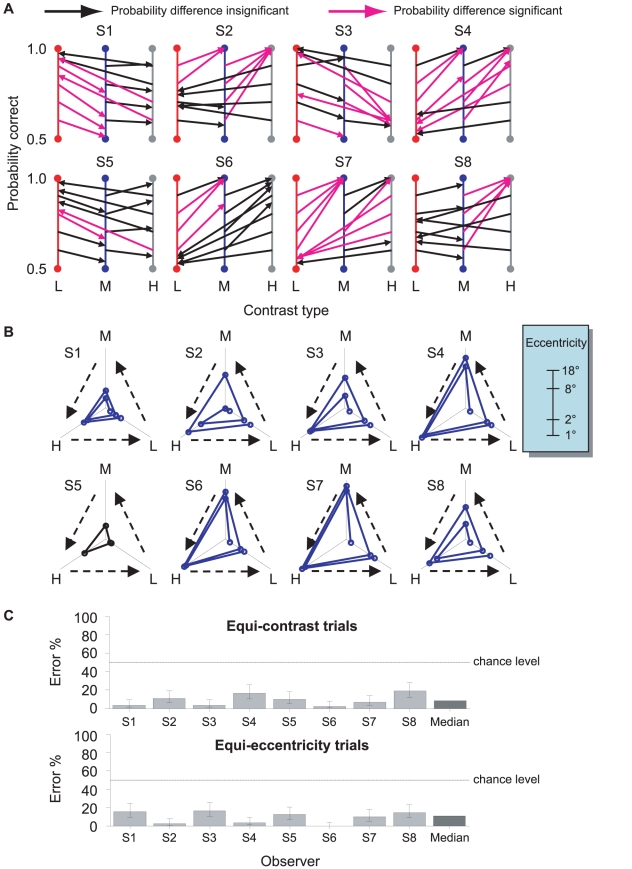
Decision task: Results of the three tests. **A. Equivalence.** For each observer, we estimated 12 eccentricity-contrast pairs 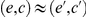 that the observer judged to be equally discriminable. We compare observers' judgments to actual discrimination performance for that observer measured in the calibration task. Suppose, for example, that an observer judges 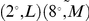, that is, a low contrast target at 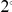 eccentricity is as discriminable as a medium contrast target at 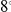. Based on calibration performance, we estimate that probability correct for 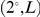 was 0.93 while that for 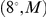 was 0.61. We plot these probabilities on the vertical colored axes for L (red) and M (blue) and connect them by a straight line with an arrow at the end corresponding to the eccentricity-contrast pair whose eccentricity varied in the staircase procedure. If the line segment is horizontal then the observer correctly judged the pairs to be equally discriminable. If the line segment is significantly slanted, the observer is in error. We plotted each of the 12 pairs judged equally discriminable (four for each possible pair of contrasts) in this way. The labels L, M, and H, or the colors red, blue, gray, respectively denote low, medium, and high contrasts. Black denotes an insignificant probability difference while magenta denotes a significant probability difference. The overall significance level is .05, Bonferroni corrected for 12 conditions (that is, each test had a size of .0042 = .05/12). The observers' judgments exhibit large, patterned failures. **B. Transitivity.** Each panel corresponds to one observer. The three axes of the inverse Y configuration are for the three contrasts. L, M, H denotes low, medium, and high contrasts, respectively. On each axis, the distance of a point to the center represents the eccentricity of a target, ranging from 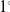 to 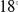 on a log scale (see inset). Lines connect eccentricity-contrast pairs of subjective indifference. For each observer, we started from 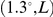, used the low-to-medium equivalence transformation to locate the eccentricity 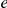 for 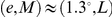, and then used the medium-to-high mapping to move to the next and so on. If the low-to-medium, medium-to-high, and high-to-low mappings satisfy transitivity, the fourth point should fall on the starting point. A gap between them implies intransitivity. Observers that significantly failed the transitivity test are plotted in blue with six mapping lines. The observer that passed the transitivity test is plotted in black ending at the third mapping line. Notice that all the intransitive mapping lines spiral outward from the center. See text. **C. Dominance.** Percentage of errors for each observer and their median in the equi-contrast trials (top) and equi-eccentricity trials (bottom). Error bar denotes the 95% confidence interval. Dashed lines mark the chance levels.

Figure 4A shows the differences of probability correct in each staircase separately for each observer. The vertical axis denotes probability correct. Each arrowed line points from the fixed target to the variable target. If a subjective indifference pair has identical probability correct for the fixed and variable targets, the arrowed line should be horizontal. Slanted lines correspond to differences in probability. Pairs with significant probability difference are in magenta.

We noticed that observers' errors were not random in direction. In Figure 4A, the magenta lines for the same observer deviate from the vertical orientation either clockwise or counter-clockwise, but never in both ways. This pattern is an indication that the observer consistently overestimated or consistently underestimated the effect of differences in contrast on visual sensitivity.

To verify this claim we computed probability difference of the lower contrast target minus the higher contrast target averaged across the 12 subjective indifference pairs for each observer. According to two-tailed Student's *t*-tests, all observers' mean probability difference was significantly different from zero (*p*<.05). Among the eight observers, three overestimated the visual sensitivity difference and the other five underestimated it.

We also measured observers' errors in eccentricity. The correct eccentricity of the variable target in a staircase was defined as the eccentricity where the variable target had the same probability correct as the fixed target. Eccentricity error of a subjective indifference pair was the actual eccentricity of the variable target minus the correct eccentricity. The absolute error averaged across the 12 pairs was 1.6, 5.8, 1.9, 7.5, 2.2, 7.7, 6.4, and 5.0 degrees respectively for S1–S8. Their median was 5.4 degrees. Therefore, observers' errors in the decision task were unlikely to be a byproduct of lack of ability to discriminate eccentricity.

### Transitivity test

In the equivalence test, we tested whether observers made judgments consistent with their actual ability to classify stimuli differing in contrast and eccentricity. They did not do so. Next we examined whether observers' judgments, even though in error, were self-consistent by testing transitivity (one of the necessary conditions for a conjoint measurement representation) as follows. An observer's judgments are transitive if and only if, for all choices of eccentricities 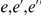 and contrasts 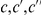: if 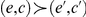 and 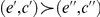, then 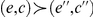. We test transitivity in a slightly different form by measuring eccentricity-contrast pairs 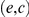, 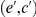 that are judged equally discriminable. We denote equal discriminablity as 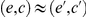. We test the following: if 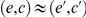 and 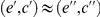, then 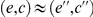.

Suppose the function 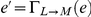 transforms any eccentricity 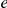 at low contrast into an eccentricity 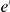 at medium contrast that the observer judges to be equally discriminable. That is, the observer, rightly or wrongly, judges 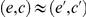. We refer to 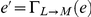 as an *equivalence transformation*.

From the decision task, we can estimate the equivalence transformations of low-to-medium, medium-to-high, and high-to-low contrasts, respectively denoted as 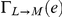, 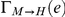, and 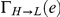. The criterion for transitivity is that transitivity holds, 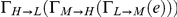 should transform 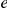 back to 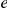, that is,
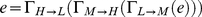
(2)


In our transitivity test, we assume that the subjective probability correct for a particular contrast, like the true probability correct, is a function of eccentricity in the form of Equation 1. An equivalence transformation from one contrast to another contrast would then be linear on a log scale (see Text S1 for proof).
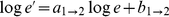
(3)where 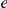 is the eccentricity for contrast 1, 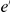 is the eccentricity for contrast 2, and 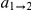 and 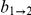 are parameters to be estimated.

If Equation 2 is satisfied, we should have
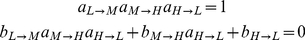
(4)Define 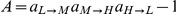, 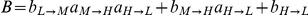. Testing for failure of transitivity requires only that we test whether either of 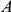 and 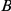 is significantly different from zero.

For each observer, we fitted Equation 3 separately for the low-to-medium, medium-to-high, and high-to-low transformations. With the estimated 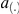
's and 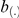
's we computed 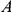 and 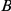. We obtained the 95% confidence intervals (Bonferroni corrected for two conditions) of 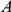 and 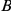 using a bootstrap method [18] by resampling the staircase data for 10,000 times.

Only one observer (S5) passed the transitivity test. The remaining seven observers' mean 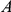 and 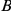 values were both significantly different from zero. Interesting, all the seven observers' deviations had the same direction. That is, all the 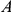
's were less than zero (median across observers = −0.60). All the 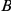
's were greater than zero (median across observers = 1.06). If, for any observer, 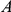 and 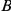 errors were independent and equally often positive or negative, the probability for all the seven observers to have a less-than-zero 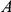 and a greater-than-zero 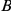 would be 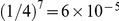. Therefore, the observed common pattern of failure of transitivity is unlikely to be the result of measurement error.

Figure 4B shows a sequence of transformations. The three axes in each subplot represent the eccentricities of the low, medium, and high contrasts in the log scale. For each observer, we start from a specific eccentricity at the low contrast find the equivalent eccentricity at the medium contrast, then we pass from medium to high and then high to low. If the transformations satisfy transitivity, we should return to the same eccentricity at the low contrast axis after going through the three transformations, low-to-medium, medium-to-high and high-to-low. If transitivity holds, we stop after one set of three transformations (low→medium→high→low). If it does not we continue with a second set of three transformations to make the pattern of intransitivity easier to visualize.

Figure 4B illustrates the transitivity failure of seven out of eight observers and their common pattern of failure. We move from one axis to another axis in an arbitrary counter-clockwise way. Note that all the observers that failed the transitivity test had plots that tended to “corkscrew” outward. That is, when eccentricity is transformed from low contrast to medium contrast and then to high contrast, the resulting eccentricity difference between the low and high contrasts tended to be larger than when they mapped from low to high directly.

### Dominance test

Observers failed the equivalence test and, with one exception, the transitivity test. The dominance test is, in conjoint measurement terms, a test that observer's preferences form a weak order satisfying single cancellation [15]. We are asking whether observers, given two targets of equal contrast at different eccentricities, judge the target with smaller eccentricity to be more discriminable (equi-contrast dominance) and that, given two targets at the same eccentricity, judge the target with higher contrast to be more discriminable (equi-eccentricity dominance).

Figure 4C show the percentage of dominance errors for each observer. For each observer and condition, we computed the 95% confidence intervals for the percentage of errors by treating the true proportion of errors as a random variable with a beta distribution whose parameters are determined by the observed numbers of errors and non-errors. Although the percentage of errors was significantly larger than zero for most of the observers in either condition, the values were small. The medians across observers were 8.3% and 11%, respectively for the equi-contrast trials and equi-eccentricity trials. The upper limits of all the confidence intervals were far below 50%, the chance level.

## Discussion

We employed a simple decision task with a conjoint measurement design to investigate what people know about their own visual uncertainty across the retina. In this task, observers were asked to judge which of two eccentricity-contrast pairs 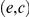 or 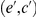 was more discriminable. We measured the observer's ability to discriminate targets varying in contrast and eccentricity separately in a calibration task. Consequently, we could determine whether the observer correctly judged which of the two eccentricity-contrast pairs was more discriminable. We found that observers' judgments exhibited large, patterned errors.

Observers may err in judging equally-discriminable pairs, but be self-consistent in their erroneous judgments. We tested whether observers' judgments were transitive. An observer's judgments are transitive if and only if, for all choices of eccentricities 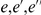 and contrasts 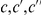: 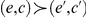 and 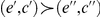, then 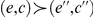. Seven out of eight observers failed to be transitive, exhibiting large and patterned errors.

The last test, dominance, assessed whether the observer would choose the eccentricity-contrast pair with smaller eccentricity if contrasts were the same or the eccentricity-contrast pair with larger contrast if eccentricities were equated. An observer need only have a crude sense that higher contrast leads to better performance and that performance is better at smaller eccentricities, at least for our choice of stimuli. In particular, an observer can “pass” dominance without any ability to trade off the consequences of differences in contrast with differences in eccentricity. We found significant failures of dominance but the rate of failure was small and observers were far from the chance level that would indicate a complete failure of dominance.

The observer maximizes his expected gain by always choosing the contrast-sensitivity pair that is more readily detectible. If the probability of detection of one pair is 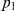 and that of the second is 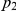 and 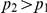 then the observer should choose the second. If he chooses the first then he reduces his winnings by 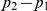 multiplied by the reward received for a correct response. The consequence of failures of equivalence, transitivity and dominance, taken together, was to reduce the expected winnings of all observers by between 10% and 18%.

Previous work amply demonstrates that memory for the location of targets decays significantly over time [19]. However, in our experiment each target location were marked by a gray square present throughout the trials and the discrimination task was to judge the location of a white dot relative to the gray square. Consequently, observers' poor performance in the choice task is unlikely to be due to any uncertainty concerning the location where the target would appear.

In conclusion, we find little evidence that observers can accurately assess their visual sensitivity or even order eccentricity-contrast pairs consistently. Their consistent patterns of transitivity violation suggest that contrast and eccentricity are treated as two dimensions that constitute a lexicographic semiorder [20].

We emphasize that the decision task inducing these failures in judgment was remarkably simple. Since rewards never varied, failures to judge probability correctly could not be due to assignment of subjective utilities to rewards. Moreover, any invertible distortion of probability of the sort commonly found in the decision making literature [12] would not affect performance in the task at all.

Previous work in cue combination and in motor planning suggest that the human visuo-motor system has access to estimates of its own visuo-motor uncertainty in various tasks [21]–[22]. Human ability to anticipate performance in simple visuo-motor tasks is well documented. Observers with comparable training in motor tasks do learn and compensate for their own visuo-motor variability in later pointing tasks [23]–[24] and can accurately estimate their chances of success when shown a rectangular target and asked to estimate the chances they could hit it [25]. Observers can also rapidly select which of two pointing target configurations has higher expected value in a task analogous to ours [26]. In all these experiments observers considered multiple hypothetical actions and, without further practice or feedback, selected actions that maximized expected gain, or nearly so.

We consider a very similar task but in a different domain: judgments of retinal sensitivity as a function of contrast and eccentricity. In contrast to performance in these visual and motor tasks, however, our observers not only had markedly distorted representations of their retinal scaling functions for targets differing in contrast but also made choices that violated transitivity.

The observed failure to correctly judge visual sensitivity across retinal positions agrees well with reports in other areas. Patients suffering from scotomas (a retinal area with reduced visual acuity) are typically unaware of the scotoma even when testing reveals near total loss of visual sensitivity outside the fovea [27]. Most patients with a central scotoma prefer to use their left visual field to read, although the right visual field is found to be more efficient in reading than the left visual field [28]–[29]. Galvin & Williams [30] noted that, while objective visual performance in many tasks plummets with distance from the fovea, human observers seem to experience a retinal field that is unblurred and more or less uniform. People were found to have underconfidence and overconfidence at the same time for visual discrimination performance of stimuli of different size [31].

Our results suggest that people might have difficulty in integrating the uncertain visual information from across different retinal eccentricities to speed search. In fact, people are reported to be suboptimal at choosing where to saccade [32]–[35].

One possibility is that observers have inaccurate estimates of retinal eccentricity [36]–[37], which make precise mapping between eccentricity and probability of correct impossible. But the observers' transitivity failures suggest the failures are more profound: people likely do not have consistent estimates of visual sensitivity at all.

### 

#### Heuristic-based visual search

Our results are in apparent conflict with the results of Najemnik & Geisler [7], [9]–[10], which show good human performance in selection of saccades in visual search. One possibility is that the visual system has accurate information concerning visual sensitivity to different targets as a function of eccentricity but that this information is unavailable for the sort of comparative judgments we considered here.

But a second possibility is that human visual search is actually based on simple heuristics plus a qualitative understanding of one's visual sensitivity map. Such a heuristic-based approach may approximate ideal performance in some tasks while failing utterly in others. The experimenter who considers performance in a limited range of scenes may record behavior that approximates optimal but is in fact no more than a lucky coincidence of a heuristic rule and experimental conditions. Such “apparent optimality” is not rare in behavioral studies of animals [38] or humans [39].

The task used by Najemnik & Geisler [7], [9]–[10] involved detection of a Gabor patch in a 1/f field of noise and only the overall pattern of saccades was considered in evaluating the model. In contrast, we designed our simple task so that the visual system can *only* succeed if it has access to estimates of visual sensitivity for the range of contrasts and eccentricities we considered.

Dominance was the only test where subjects predominantly succeeded and their success could be due to a preference for higher contrast [34] or a preference for locations closer to the fovea [32]. According to the errors in the equivalence test, among the seven observers that failed transitivity, five observers' decisions could result from a bias toward selecting the nearer target. In the context of eye movements, this bias would correspond to a preference for shorter saccades over longer.

We conjecture that this preference for short saccades could be an oculo-motor heuristic serving to integrate the visual sensitivity map into saccade selection. A key prediction of Najemnik & Geisler's model [7], [9]–[10] is exactly that observers will prefer short saccades. Tatler and Vincent [40] presented compelling evidence that saccade selection could be better predicted by oculo-motor preferences than by visual information or task.

If human saccade decisions are based on such heuristics rather than on a computation that requires knowledge of visual sensitivity maps, we would expect a failure of adjustment when one's visual sensitivity map is changed. In fact, when observers' foveae were artificially shifted with gaze-contingent techniques, their performances in visual search were significantly worse than predicted by the ideal-observer model [41].

There is evidence that saccade selection and explicit perceptual decision in visual search pick the same location [42]–[43]. This was previously understood as evidence that saccade selection and explicit decision use the visual sensitivity map in the same way. However, if humans have little knowledge of their own visual sensitivity map, as our results suggest, and their saccades are chosen through oculo-motor heuristics, it might be their explicit decision actually comes from their saccade behavior. If so, the observer might have no way of finding out which combination of eccentricity and contrast offered the higher probability of successful detection than by observing his preferences among potential saccades, something like deciding what to have for lunch by waiting to see which sandwich your hand selects.

## Supporting Information

Text S1 Proof for Equation 3.
(0.06 MB DOC)
