# Supplementary material for: Gambling in the Visual Periphery: A Conjoint-Measurement Analysis of Human Ability to Judge Visual Uncertainty
Source: PLoS Comput Biol. 2010 Dec 2;6(12):e1001023. doi: 10.1371/journal.pcbi.1001023 (PMC2996320; doi:10.1371/journal.pcbi.1001023)
Supplement: Text S1 — Proof for Equation 3. (0.06 MB DOC) [file pcbi.1001023.s001.doc]

**Text S1**

Suppose $\psi_{1}\left(e\right)$ and $\psi_{2}\left(e\right)$, both in the form of Equation 1 but with different parameters, are the probabilities correct at eccentricity $e$ for each of Contrast 1 and 2, respectively. Suppose that eccentricity $e_{1}$ at Contrast 1 and eccentricity $e_{2}$ at Contrast 2 lead to the same probability correct. That is, $\psi_{1}\left(e_{1}\right)=\psi_{2}\left(e_{2}\right)$. Substitute $\psi_{1}\left(e\right)$ and $\psi_{2}\left(e\right)$ from Equation 1, we get

$0.5+0.5\exp\left(-\left(e_{1}/\alpha_{1}\right)^{\beta_{1}}\right)=0.5+0.5\exp\left(-\left(e_{2}/\alpha_{2}\right)^{\beta_{2}}\right)$.

If we cancel common constants and take the natural logarithm of both sides, the equation becomes

$$\beta_{1}\left(\log e_{1}-\log\alpha_{1}\right)=\beta_{2}\left(\log e_{2}-\log\alpha_{2}\right)$$

Suppose that the observer also has the same probability correct for Contrast 1 at eccentricity $e'_{1}$ and Contrast 2 at eccentricity $e'_{2}$. Then

$$\beta_{1}\left(\log e'_{1}-\log\alpha_{1}\right)=\beta_{2}\left(\log e'_{2}-\log\alpha_{2}\right)$$

Subtracting this equation from the previous equation, we obtain a linear relation between the logarithms of eccentricities:

$$\beta_{1}\left(\log e_{1}-\log e'_{1}\right)=\beta_{2}\left(\log e_{2}-\log e'_{2}\right)$$

Let $a_{1\to2}=\frac{\beta_{1}}{\beta_{2}}$, $b_{1\to2}=\log e'_{2}-\frac{\beta_{1}}{\beta_{2}}\log e'_{1}$, the equivalence transformation from Contrast 1 to Contrast 2 could then be written as

$$\log e_{2}=a_{1\to2}\log e_{1}+b_{1\to2}$$
